# MiR-22-3p suppresses NSCLC cell migration and EMT via targeting RAC1 expression

**DOI:** 10.1007/s10142-023-01211-z

**Published:** 2023-08-25

**Authors:** Xuejiao Wang, Xiaobin Wang, Tao Jiang, Zhipei Zhang, Nianlin Xie, Guang Yang

**Affiliations:** Department of Thoracic Surgery, The Second Affiliated Hospital of the Air Force Medical University, Baqiao District, Xinsi Road 569, Xi’an, Shaanxi China

**Keywords:** miR-22-3p, Lung cancer, RAC1, Migration, EMT, Luciferase assay

## Abstract

Previous studies have demonstrated the tumor-suppressive function of microRNA-22-3p (miR-22-3p) in several cancers, whereas the significance of miR-22-3p in non-small cell lung cancer (NSCLC) remains unclear. In this study, we explored the biological function and molecular mechanism of miR-22-3p in NSCLC cells. First, we assessed the expression of miR-22-3p in NSCLC tissues and cells based on RT-qPCR and TCGA database. Compared with normal lung tissues and cells, miR-22-3p expression was dramatically decreased in lung cancer tissues and cells. miR-22-3p expression was also correlated with lymph node metastasis and tumor size, but not TNM stages. We further explored the in vitro function of miR-22-3p on the migration and epithelial–mesenchymal transition (EMT) of NSCLC cells. The results showed that overexpression of miR-22-3p suppressed the migration and EMT of NSCLC cells, whereas silencing miR-22-3p showed the opposite effect. Luciferase assay demonstrated that RAS-related C3 botulinum toxin substrate 1 (RAC1) was the target gene for miR-22-3p. Mechanistically, we demonstrated that miR-22-3p suppressed the cell migration and EMT via downregulation of RAC1 because the inhibitory effect of miR-22-3p on cell migration and EMT of NSCLC cells was reversed by RAC1 overexpression. Based on these novel data, the miR-22-3p/RAC1 axis may be an alternative target in the therapeutic intervention of NSCLC.

## Introduction

Lung cancer is the second most commonly diagnosed cancer worldwide and was the leading cause of cancer-related deaths in 2020, with approximately 2.2 million new cancer cases (11.4%) and 1.8 million deaths (18%) (Sung et al. 2021). The main cause of lung carcinogenesis is tobacco consumption, followed by other etiological factors like occupational exposures, air pollution, poor diet, and genetic susceptibility; these may be individual risk factors or combined with cigarette smoking in shaping the descriptive epidemiology of lung cancer (Malhotra et al. 2016). Approximately 80% of lung cancer cases are non-small cell lung carcinomas (NSCLCs), while the remaining ones are small cell lung cancers (SCLCs). Recently, many revolutionary advances have been made in managing lung cancer in screening, diagnosis, and therapy using low-dose CT screening, immunotherapy, and molecular-targeted therapy (Alexander et al. 2020). The estimated 5-year survival rate of lung cancer patients diagnosed in 2010–2014 ranged between 10 and 30% in 34 countries, and the survival increased by more than 10% in China (Allemani et al. 2018).

miRNAs are small non-coding RNAs that regulate the expression of thousands of genes post-transcriptionally in normal physiological or disease contexts (Pritchard et al. 2012). miRNA expression profiling has been found to be related to tumor progression, development, and therapeutic reaction, which suggests their probable application as predictive, diagnostic, and prognostic biomarkers (Iorio and Croce 2012). In lung cancer, increasing research indicates that miRNAs are critical in modifying the microenvironment, enhancing tumor progression, angiogenesis, metastasis, invasion, and drug resistance (Wu et al. 2019; Hu et al. 2020). For example, microRNA-1246 was down-regulated and suppressed the invasion of NSCLC cells through negative regulation of CXCR4 (Xu et al. 2018). Another study identified that microRNA-218 down-regulation was associated with the worse prognosis of NSCLC patients. Silencing of microRNA-218 contributed to the enhanced the progression of NSCLC cells in vitro and in vivo by repressing IL-6 receptor (Yang et al. 2017). In contrast to the function of microRNA-1246 and microRNA-218, miR-21 functions as a tumor-promoting microRNA in NSCLC. miR-21-5p/SMAD7 axis promoted the progression of NSCLC (Tang et al. 2021). These evidences suggest that dysregulation of microRNAs play an essential role in NSCLC development.

According to various reports, miR-22-3p serves as a tumor suppressor in several cancers including breast (Wang et al. 2021), glioblastoma (Ma et al. 2021a), bladder (Tian et al. 2021), colorectal (Wang and Lin 2021), gastric (Zhang et al. 2020), hepatocellular (Chen et al. 2016), cervical (Lv et al. 2018), and osteosarcoma (Xue et al. 2022). In lung cancer, Ma et al. (Ma et al. 2021b) reported decreased miR-22-3p expression, correlating with patients’ prognosis of lung adenocarcinoma and clinicopathological characteristics. Yang et al. (Yang et al. 2021) found that miR-22-3p suppressed lung cancer cell growth through MET/STAT3 signaling. In addition, Jiang et al. (Jiang et al. 2019) revealed that miR-22-3p strengthened the radiosensitivity of small cell lung cancers via targeting WRNIP1. Furthermore, Zhang et al. (Zhang et al. 2017) showed that miR-22 inhibited lung cancer cell EMT and invasion through targeting snail. Targets of miR-22-3p involved in lung cancer progression included yes-associated protein (YAP1) (He et al. 2020), MET (Yang et al. 2021), ATP citrate lyase (ACLY) (Xin et al. 2016), enolase1 (ENO1) (Zhou et al. 2019), and MALAT1 (Li et al. 2020). These studies demonstrate the crucial role of miR-22-3p during malignant tumor progression. Targeting the substrates of miR-22-3p might be helpful for the treatment of cancer patients with lowly expressed miR-22-3p. Although numbers of targets of miR-22-3p have been identified by various studies, none of the drugs or inhibitors against these targets is under consideration in clinical studies. Thus, discovering novel substrates of miR-22-3p in malignant cancer, such as NSCLC, might help the pharmaceuticals companies developing effective drugs to treat the malignant cancers triggered by down-regulation of miR-22-3p.

In this study, we aimed to explore the clinical relevance, biology function, and the downstream targets of miR-22-3p in NSCLC. Our findings highlight a new axis of miR-22-3p/RAS-related C3 botulinum toxin substrate 1 (RAC1) involved in lung cancer cell migration and EMT.

## Materials and methods

### Samples from The Genome Cancer Atlas (TCGA) and clinical patients

We collected a total of 23 pairs of NSCLC tissue samples and matched normal tissues from the Department of Thoracic Surgery in the Second Affiliated Hospital, the Air Force Medical University, which was authorized by the Ethics Committee of the Second Affiliated Hospital, the Air Force Medical University (ethical approval number was TDLL-202304–03). We analyzed clinical data of the 23 pairs of NSCLC samples to survey the relationship between miR-22-3p expression level and clinicopathological characteristics (including smoking history, age, tumor differentiation, gender, tumor size, lymphatic metastasis, and TNM classification). To further explore the clinical significance of miR-22-3p in NSCLC from TCGA data, we analyzed the expression miR-22-3p in NSCLC and normal samples according to the dataset of ENCORI (https://starbase.sysu.edu.cn/panCancer.php). A total of 1085 cancer samples and 104 normal tissues derived from NSCLC patients were included in this study.

### Cell culture and transfection

We acquired the human NSCLC cell lines A549 and Calu-1, and the bronchial epithelial cell line BEAS-2B, from the American Type Culture Collection (ATCC, Manassas, VA, USA), with cells cultured in DMEM/F-12 medium (Gibco, Grand Island, NY, USA) supplemented with 10% fetal bovine serum (FBS; Gibco, Grand Island, NY, USA) and 100 U/mL penicillin/streptomycin (Sigma, USA) in a 5% CO_2_ incubator at 37 °C.

We synthesized mimics-NC (5′-UUUGUACUACACAAAAGUACUG-3′), miR-22-3p inhibitor (5′-ACAGUUCUUCAACUGGCAGCUU-3′), miR‐22-3p mimics (5′-AAGCUGCCAGUUGAAGAACUGU-3′), inhibitor-NC (5′-CAGUACUUUUGUGUAGUACAAA-3’) using HippoBiotec (Huzhou, China), and cloned ORF of RAC1 (NM_006908.5) into pcDNA3.1 vector (Promega) for overexpression of RAC1. Cell transfection was done using Lipofectamine 2000 (Invitrogen) as per the manufacturer’s protocol. Two days later, the cells were subjected to other experiments.

### Real-time quantitative polymerase chain reaction (RT-qPCR)

Total RNA from tissues samples or transfected cells was extracted using RNAiso Plus Kit (TaKaRa, Japan) as per the manufacturer’s guidelines. The concentration of total RNA was determined by using the NanoDrop. We adopted SuperScript III® (Invitrogen) for cDNA amplification. qPCR analysis was carried out using an AceQ RT-qPCR Kit (Vazyme) on a BioRad CFX96 Sequence Detection System (BioRad company, Berkeley, CA, USA). The cycling condition was described as follows: step 1, holding stage, 95 ℃ for 3 min; step 2, cycling stage, 95 ℃ for 15 s; step 3, cycling stage, 60 ℃ for 30 s; step 4, cycling stage, 72 ℃ for 30 s; step 2 to step 4, 40 cycles. Primer of miR-22-3p and U6 was purchased from Ruibo (Guangzhou). Primer sequences of RAC1 and β-actin were as follows: RAC1, forward, 5′-ATGTCCGTGCAAAGTGGTATC-3′, reverse, 5′-CTCGGATCGCTTCGTCAAACA-3′; β-actin, forward, 5′-CATGTACGTTGCTATCCAGGC-3′, reverse, 5′-CTCCTTAATGTCACGCACGAT-3′. We normalized mRNA levels using U6 and β-actin. The relative expression of miR-22-3p or RAC1 was analyzed using the 2 − ΔΔCt method.

### Wound healing assay

We tested the migratory capacity of cells using wound healing assays. Transfected cells were cultured in six-well plates for 1 day. Then, a 10-μL pipette was used to scratch a wound into the middle part of each well, followed by a change of medium to 1% FBS-containing DMEM medium. Photos of the wound at 0 and 24 h were taken. The migration percentage was then calculated using Image Pro Plus software (IPP; produced by Media Cybernetics Corporation, USA).

### Transwell assay

Transwell cell migration assays were conducted using 24-well transwell chambers (Costar, San Diego, CA, USA). Briefly, transfected cells in non-serum medium with a density of 1 × 10^5^ cells per 500 µL were placed in the upper chambers. In the lower chambers, 500 µL of absolute DMEM medium (containing 10% FBS) was added. After 2 days of incubation, the cells on the upper surface of the chambers were removed by cotton swab and migrated cells were fixed using 4% paraformaldehyde, then stained for 30 min using 1% crystal violet at normal atmospheric temperature. Invaded cells were counted using a microscope (IM 1200, Countstar).

### Western blot

Transfected cells were cultured for 2 days, then collected and lysed by vortexing with acid‐washed glass balls for 2 min, then placed on ice for 2 min; this was repeated 10 times. The supernatant was gathered after 30 min of centrifugation at 12,000 rpm, and protein concentration measured with NanoDrop (Thermo-Fisher Scientific). Protein (20 μg) was loaded onto 10% SDS-PAGE gels for 1.5 h, then transferred onto polyvinylidene difluoride membranes for 1 h, followed by blocking for 2 h in non-fat milk (5%) in Tris-buffered saline solution containing 0.1% Tween‐20, at normal atmospheric temperature. We then probed the membranes overnight at 4 °C with primary antibodies for Vimentin (1:1000, ab137321, Abcam), GAPDH (1:1000, ab9485, Abcam), N-cadherin (1:1000, ab76057, Abcam), E-cadherin (1:1000, ab231303, Abcam), β-actin (1:1000; sc-47778, Santa Cruz), and RAC1 (1:1000, ab155938, Abcam). We added horseradish peroxidase‐conjugated secondary antibody (anti‐mouse, 1:2000, Santa Cruz) for 2 h at normal atmospheric temperature and visualized protein bands using ECL chemiluminescence detection kit (Thermo-Fisher Scientific).

### Luciferase assay

TargetScan (http://www.targetscan.org), a website widely used for prediction of microRNA targets, was applied to predict the targets of miR-22-3p. Based on the results, numbers of genes were the potential targets of miR-22-3p, including ENO1, SNAIL1, and RAC1. We focused on RAC1 gene in subsequent experiments. As shown in the website, the combination point of miR‐22-3p and RAC1 3′-UTR regions is GCAGCUC and GCAGCUU. The mutation sequence of RAC1‐3′-UTR is CGUCGAC and CGTCGAA, respectively. The 3′-UTR region (WT and Mut) of the RAC1 gene were sub-cloned into pGL 3.1 luciferase reporter vector (Promega). We cultured transfected 293 T cells into 96-well plates at a density of 5 × 10^4^ cells per well, and measured luciferase activity with the Dual-Luciferase Reporter Assay System kit (Promega) on EnSpire Multimode Plate Reader (PerkinElmer, Waltham, MA, USA) according to manufacturer’s instruction, with Renilla luciferase activity as normalized control.

### Statistical analysis

All data are presented as mean ± SEM with three independent repeats. Student’ *t*-test was used to compare the difference between two groups. One-way analysis of variance (ANOVA) followed by a Tukey’s post hoc test was applied to analyze the difference among more than two groups. *P* values < 0.05 were considered statistically significant.

## Results

### miR-22-3p expression is decreased in NSCLC tissue and cells

**Fig. 1:**
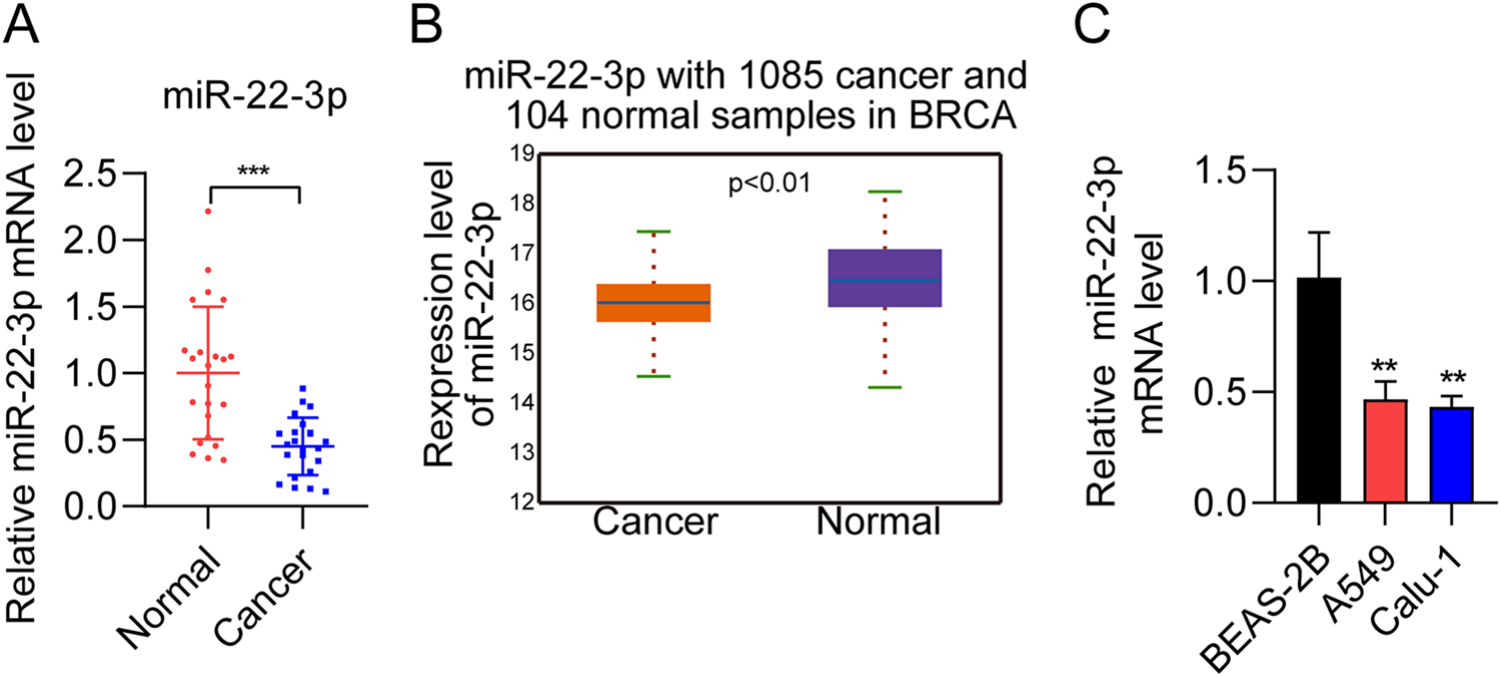
Decreased miR-22-3p expression in NSCLC tissues and cells. **A** miR-22-3p expression measured in 23 paired NSCLC and neighboring normal tissues using RT‐qPCR. **B** miR-22-3p expression measured for NSCLC and normal samples in TCGA database. **C** miR-22-3p expression measured in two NSCLC cell lines (Calu-1 and A549) and normal lung epithelial cell line (BEAS‐2B) using real-time quantitative polymerase chain reaction (RT‐qPCR). ^**^ *P* < 0.01, ^***^ *P* < 0.001

We assessed miR-22-3p expression in NSCLC tissues and cells with real-time quantitative polymerase chain reaction (RT-qPCR). We found that miR-22-3p expression in NSCLC tissues was significantly lower than that of neighboring normal tissues (Fig. 1A). miR-22-3p expression in NSCLC samples (*n* = 1085) was dramatically decreased as compared to normal samples (*n* = 104) from TCGA database (Fig. 1B). The patients’ clinical data showed that the expression of miR-22-3p influenced the patients’ lymphatic metastasis and tumor size (*P* < 0.05), but not in gender, smoking history, age, or TNM classification (Table 1). Additionally, miR-22-3p expression in lung cancer cell lines A549 and Calu-1 was detected with RT‐qPCR. The level of miR-22-3p was decreased in lung cancer cells when compared with human lung epithelial cells BEAS‐2B (Fig. 1C). These results are consistent with previous studies (Ma et al. 2021b; Zhou et al. 2019), demonstrating miR-22-3p as a potential suppressor in NSCLC.

**Table 1:** Correlation of miR-22-3p expression with clinicopathological characteristics in 23 patients of lung cancer

Variables	N	miR-22-3p expression		P Value
		High expression	Low expression	
Gender				
Male	12	6	4	
Female	11	5	6	0.505
Age				
<60	9	7	2	
>60	14	8	6	0.311
Smoking history				
Yes	10	4	6	
No	13	8	5	0.305
Tumor differentiation				
High-Middle	7	2	5	
Low	16	10	6	0.134
Tumor size				
>3	13	4	9	
<3	10	8	2	0.019
Lymphatic matastasis				
Yes	8	2	6	
No	15	11	4	0.026
TNM calassification				
I+II	9	4	5	
III+IV	14	9	5	0.349

### miR-22-3p negatively regulates the migration of lung cancer cells

**Fig. 2:**
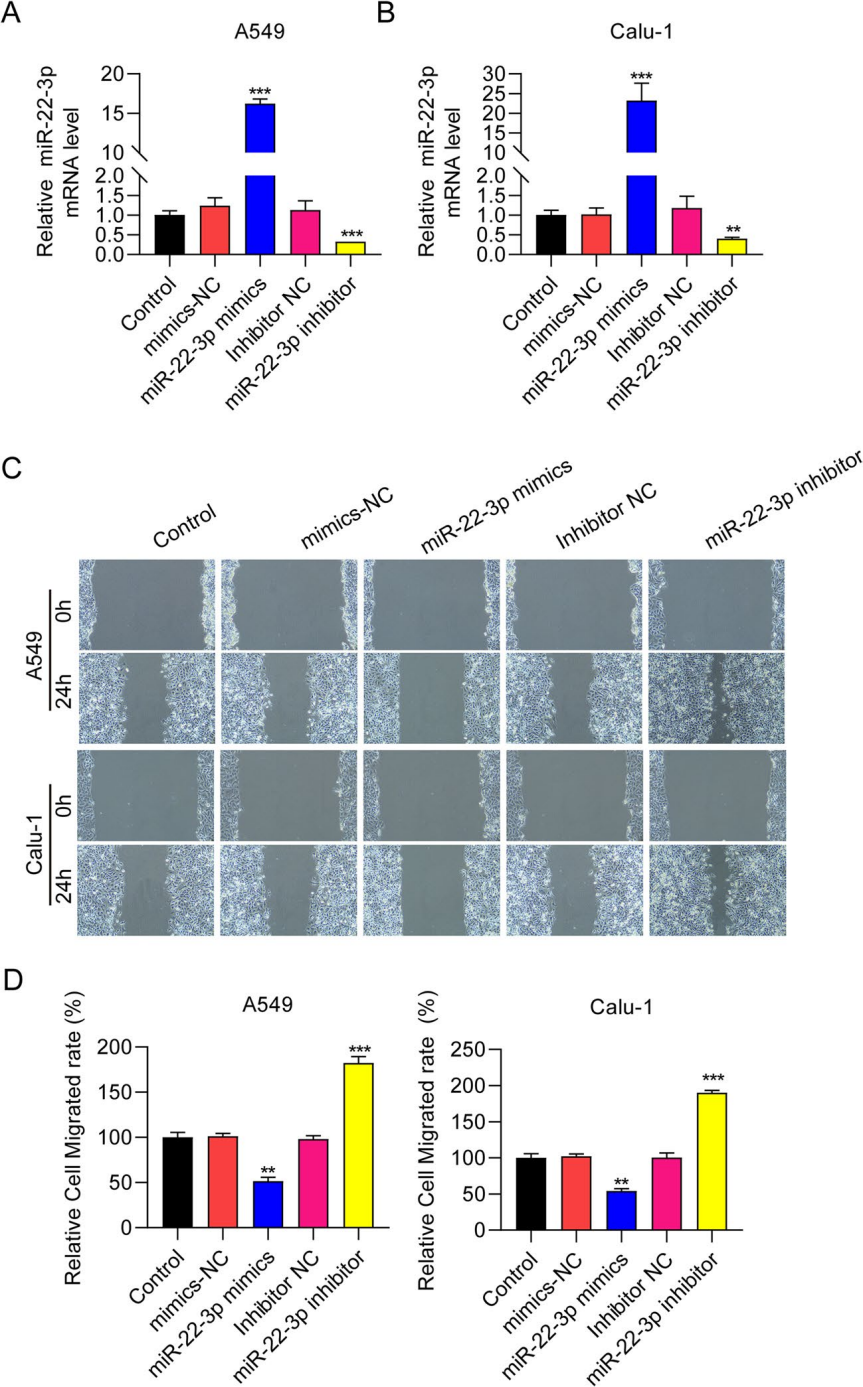
miR-22-3p negatively regulates the migration of NSCLC cells. **A**, **B** mRNA expression of miR-22-3p in Calu-1 and A549 cells transfected with control, mimics negative control (NC), miR-22-3p mimics, inhibitor NC, and miR-22-3p inhibitor. **C**, **D** Representative images and statistical analysis of cell migration of A549 and Calu-1 cells transfected with control, mimics NC, miR-22-3p mimics, inhibitor NC, and miR-22-3p inhibitor measured using wound healing assay. ^**^ *P* < 0.01, ^***^ *P* < 0.001

Both A549 and Calu-1 were NSCLC cell lines, and they had moderate level of miR-22-3p. The cell lines were suitable for overexpression and inhibition of miR-22-3p experiments. Thus, gain- and loss-of-function studies were carried out to investigate the effect of miR-22-3p on cell migration. RT-qPCR results confirmed the efficacy of miR-22-3p overexpression or knockdown in A549 and Calu-1 cells (Fig. 2A, 2B). Scratch wound assay results demonstrated that the migration of A549 and Calu-1cells was suppressed by miR-22-3p ectopic expression, whereas it was promoted by miR-22-3p silencing (Fig. 2C). The migrated cell numbers were statistically analyzed in Fig. 2D. This suggests the negative regulation of cell migration by miR-22-3p in NSCLC cells.

### miR-22-3p negatively regulated epithelial-mesenchymal transition (EMT) of NSCLC cells

**Fig. 3:**
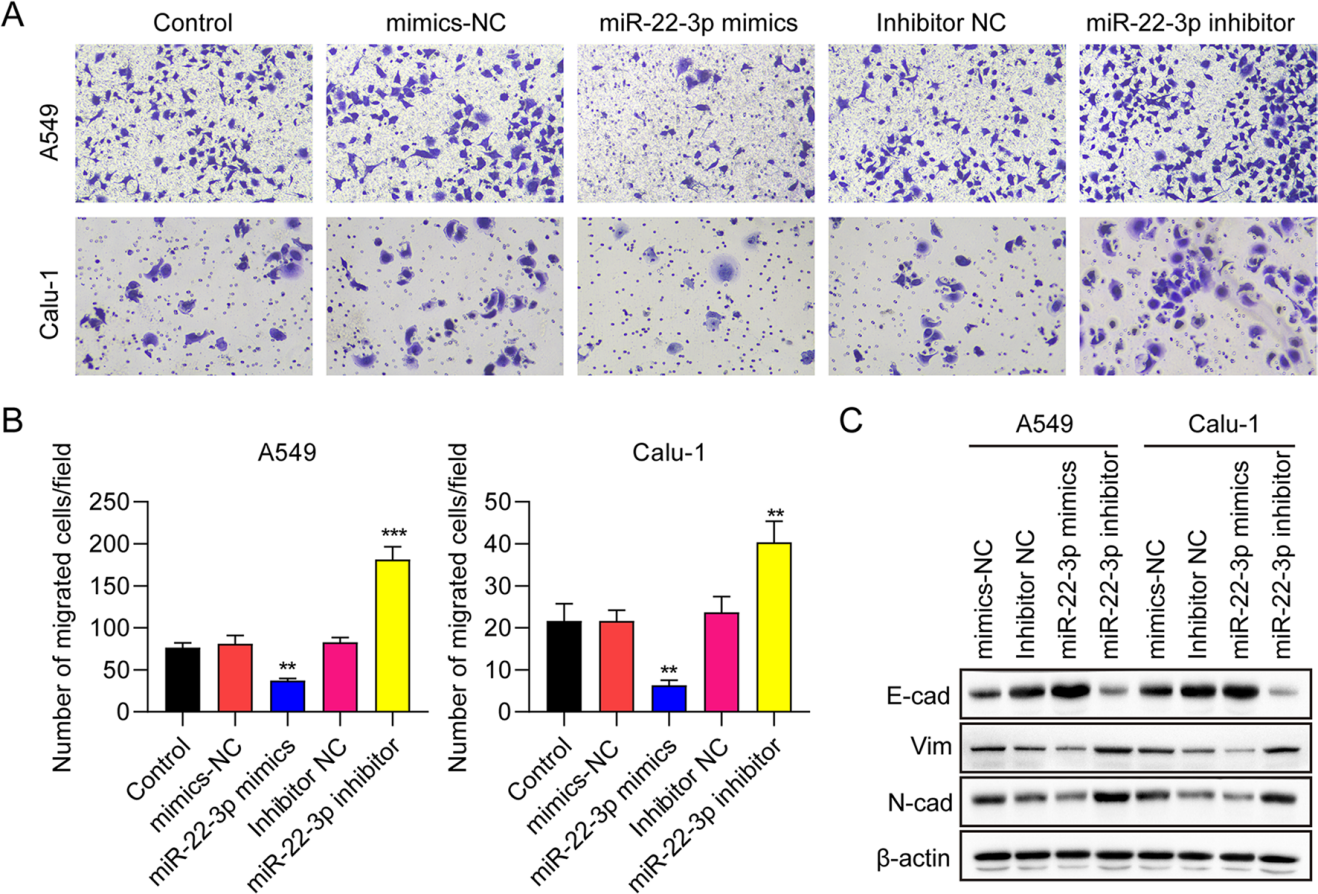
miR-22-3p suppresses epithelial-mesenchymal transition (EMT) of NSCLC cells. **A**, **B** Representative images and statistical analysis of EMT of A549 and Calu-1 cells transfected with control, mimics NC, miR-22-3p mimics, inhibitor NC, and miR-22-3p inhibitor determined by transwell assay. **C** Protein expression of E-cadherin, N-cadherin, and vimentin and in transfected A549 and Calu-1 cells measured by Western blot. ^**^ *P* < 0.01, ^***^ *P* < 0.001

In order to explore the effects of miR-22-3p on EMT in NSCLC cells, we used the transwell migration assay. We found that overexpression of miR-22-3p reduced the migration of A549 and Calu-1 cells, while silencing of miR-22-3p enhanced the migration of both cell lines (Fig. 3A). The migrated cell numbers were statistically analyzed in Fig. 3B. Western blot data demonstrated increased expression of the epithelial marker E-cadherin and decreased expression of the mesenchymal markers N-cadherin and Vimentin, after miR-22-3p overexpression. In contrast, miR-22-3p knockdown showed the opposite trend (Fig. 3C). Collectively, these results indicate that miR-22-3p negatively regulates EMT in NSCLC cells.

### miR-22-3p regulated RAS-related C3 botulinum toxin substrate 1 (RAC1) expression

**Fig. 4:**
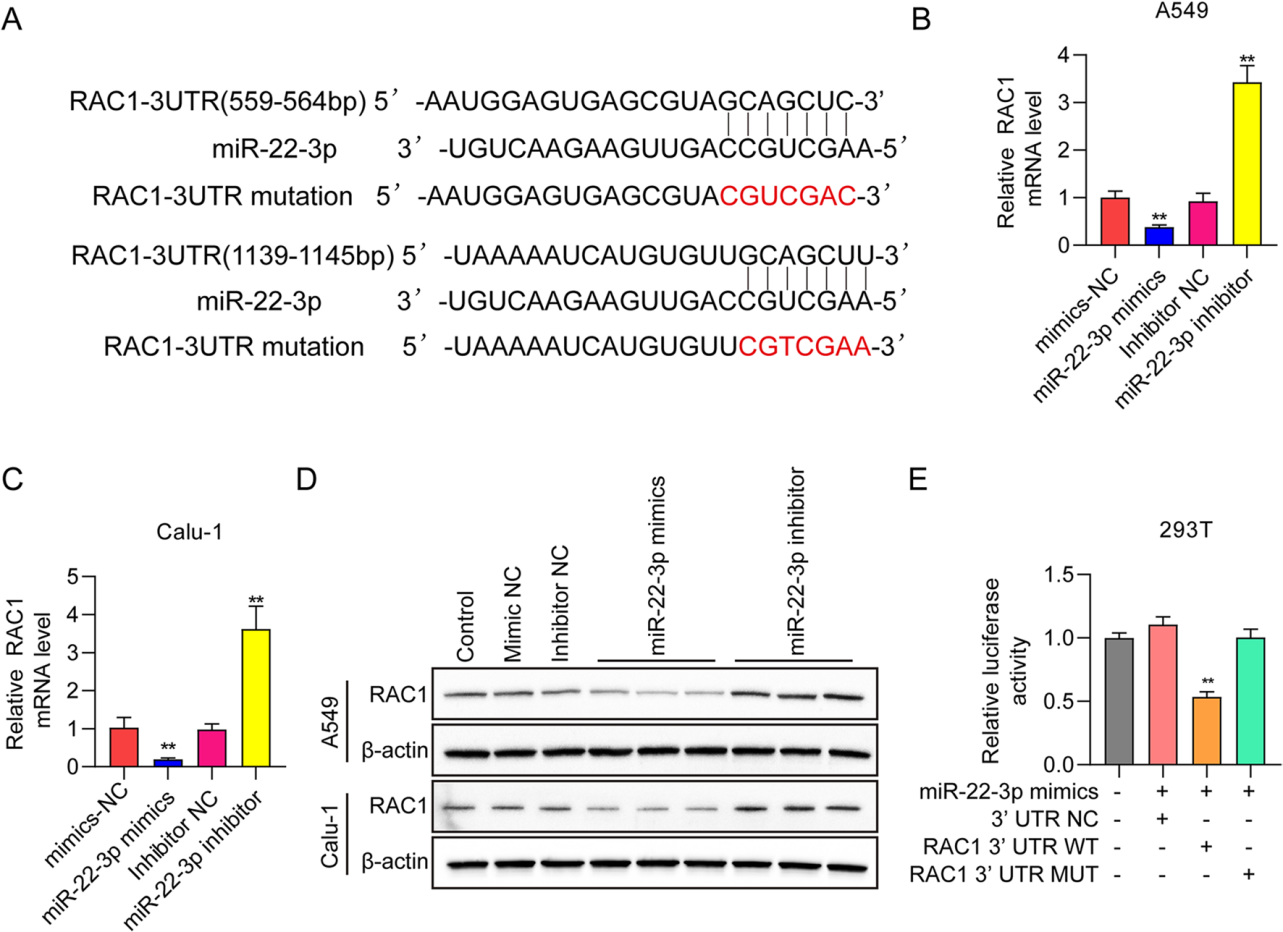
miR-22-3p inhibits RAS-related C3 botulinum toxin substrate 1 (RAC1) expression. **A** Putative miR-22-3p connecting point in 3′-UTR of RAC1 mRNA. **B**–**D** RAC1 mRNA and protein levels were detected in A549 cells and Calu-1 cell transfected using mimics NC, miR-22-3p mimics, inhibitor NC and miR-22-3p inhibitor. **E** Relative luciferase activities of reporter plasmids of 293 T cells co-transfected with miR-22-3p mimics, 3′-UTR NC, RAC1-3′-UTR-WT, or RAC1-3′-UTR-Mut. ^**^ *P* < 0.01

To investigate possible downstream gene targets of miR-22-3p, the bioinformatics tool TargetScan was adopted to predict putative targets, discovering the 3′-UTR of RAC1 gene (Fig. 4A). Based on this, the relationship between RAC1 and miR-22-3p expression in lung cancer cell lines was studied. Seen from RT-qPCR results, the mRNA levels of RAC1 were dramatically decreased following miR-22-3p overexpression in A549 and Calu-1 cells, whereas they were greatly increased following miR-22-3p knockdown (Fig. 4B and 4C). Moreover, RAC1 protein expression in the lung cancer cell lines was measured with Western blot. The results showed downregulated RAC1 levels in miR-22-3p overexpressed cells, and increased RAC1 levels in miR-22-3p silenced cells (Fig. 4D). Then, to generate mutant plasmids, we cloned the wild-type 3′-UTR of human RAC1 mRNA into a luciferase reporter and conducted site-directed mutagenesis. Luciferase activity of the wild-type reporter was downregulated by miR-22-3p overexpression, while the mutant reporter reversed the suppressive effect (Fig. 4E). These findings indicate that RAC1 serves as a direct substrate of miR-22-3p in NSCLC cells.

### RAC1 overexpression recovers the inhibitory effect of miR-22-3p on RAC1

**Fig. 5:**
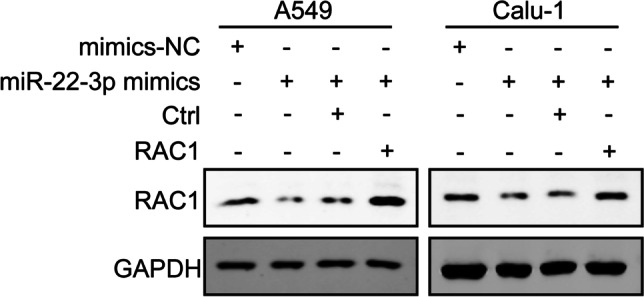
RAC1 overexpression recovers the inhibitory effect of miR-22-3p on RAC1. The immunoblotting results of RAC1 in A549 and Calu-1 cells transfected with mimics NC, miR-22-3p mimics, mimics + Ctrl and miR-22-3p mimics + RAC1-overexpressed vectors

To further explore the relationship between miR-22-3p and RAC1, we overexpressed RAC1 in A549 and Calu-1 cells transfected with miR-22-3p mimics. Western blot results showed that comparing with the miR-22-3p mimics group, protein expression of RAC1 in the miR-22-3p mimics + RAC1-overexpressed vector group was significantly increased in A549 and Calu1 cells (Fig. 5).

### miR-22-3p regulates cell migration by inhibiting RAC1 expression

**Fig. 6:**
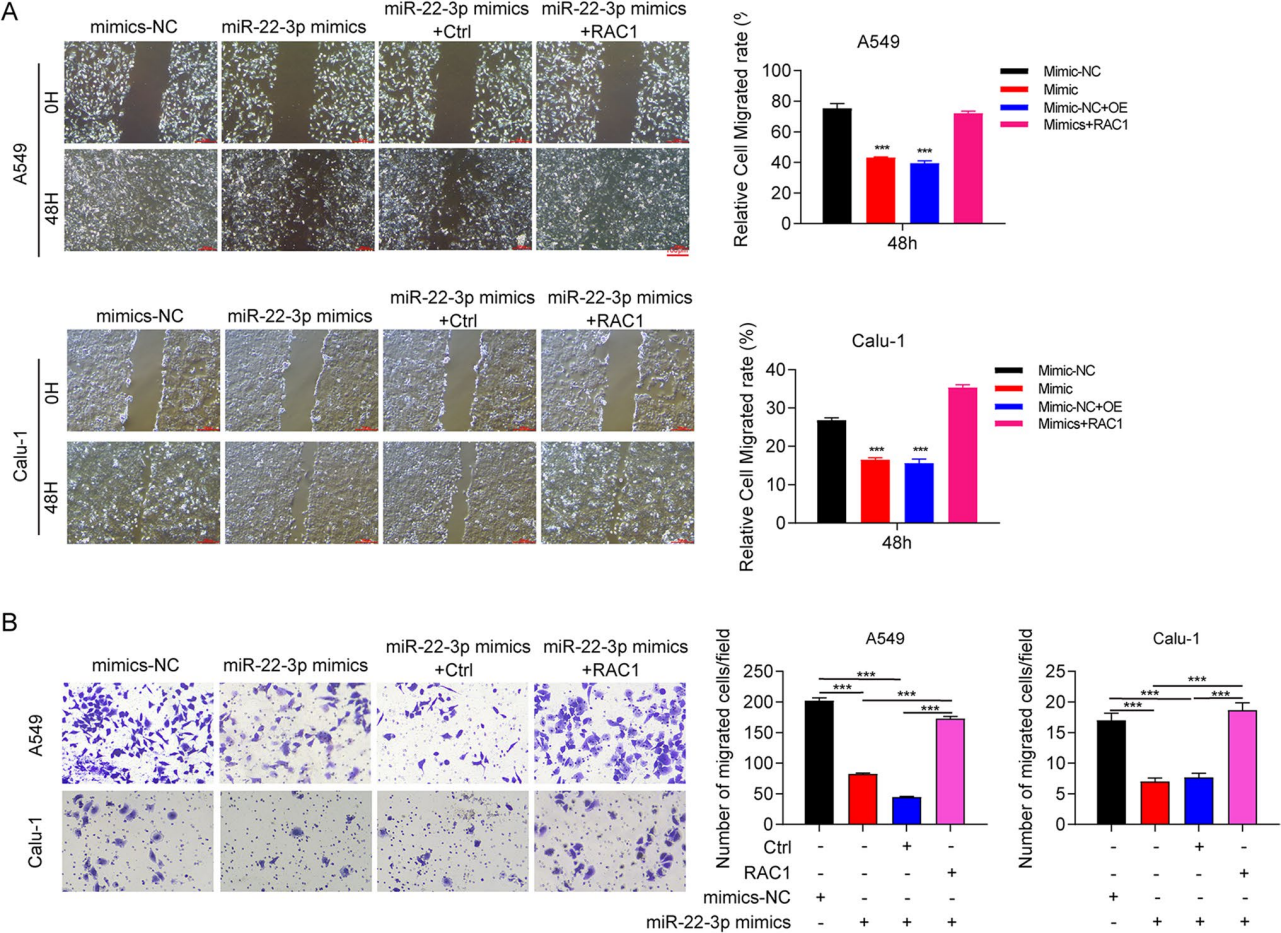
miR-22-3p suppresses the migration of NSCLC cells by inhibiting RAC1 expression. **A** Representative images and statistical analysis of cell migration of A549 and Calu-1 cells transfected with mimics NC, miR-22-3p mimics, mimics + Ctrl, and miR-22-3p mimics + RAC1-overexpressed vector measured using wound healing assay. **B** Representative images and statistical analysis of migration of A549 and Calu-1 cells measured using transwell assay. ^**^ *P* < 0.01, ^***^ *P* < 0.001

Rescue assays were carried out with EMT and cell migration experiments. A549 and Calu-1 cells were transfected with miR-22-3p mimics + RAC1-overexpressed vector, corresponding controls and mimics NC. Based on wood healing assay, miR-22-3p ectopic expression significantly repressed the invasion of A549 and Calu-1 cells, which were restored by RAC1 overexpression (Fig. 6A). Additionally, transwell assays demonstrated that overexpression of miR-22-3p caused a significant decrease in cell migration numbers in Calu-1 and A549 cells, while RAC1 ectopic expression rescued the migration ability (Fig. 6B). These results suggest that miR-22-3p inhibits NSCLC cell migration and invasion through targeting RAC1.

## Discussion

miRNAs are small, non-coding RNA molecules that regulate gene expression through direct interaction with the 3′-UTR of related target mRNAs (Kim 2005). miR-22-3p has been found to serve as a tumor suppressor in various cancers. For example, down-regulation of miR-22-3p was associated with the progression and poor prognosis of cervical cancer (Kwon et al. 2022). In breast cancer, miR-22-3p exhibited a tumor-suppressive function by targeting the expression of PLAGL2 (Fan et al. 2021). miR-22-3p was also reduced in colorectal cancer patients and inhibited the cancer malignancy through inhibition of KDM3A (Jin et al. 2022). Despite that several evidences have reported the function and potential mechanisms of miR-22-3p in lung cancer (Yang et al. 2021; He et al. 2020), the clinical significance, biology function, and especially the downstream target of miR-22-3p in NSCLC cell invasion need to be further illustrated. Here, we discovered a dramatic decrease in miR-22-3p expression in NSCLC tissues from clinical patients, lung cancer samples from TCGA dataset, and NSCLC cells compared with controls. These results are consistent with previous studies (Ma et al. 2021b). We also found that miR-22-3p expression was correlated with lymph node metastasis and tumor size, but not TNM stages. This result was consistent with the report of Ma et al. (Ma et al. 2021b) and differed from the study of Yang et al. (Yang et al. 2021), which showed that the TNM stages was also significant. And miR-22-3p overexpression also significantly suppressed the migration and epithelial-mesenchymal transition (EMT) of Calu-1 and A549 cells, regulating the expression of EMT-related proteins (vimentin, E-cadherin and N-cadherin). However, silencing of miR-22-3p exhibited the opposite effect, confirming the tumor suppressive role of miR-22-3p in NSCLC.

EMT is the biological process of changing non-motile epithelial cells into mesenchymal phenotypes with invasive capacities. The hallmark of EMT is the loss of epithelial surface markers, such as E-cadherin, and the gain of mesenchymal markers, including vimentin and N-cadherin (Na et al. 2020). Clinical studies from Grigoraş et al. indicated that reduced E-cadherin expression was related to tumoral differentiation and was associated with unfavorable diagnosis and lymph node metastasis of NSCLC patients (Grigoras et al. 2017). N-cadherin, encoded by the CDH2 gene, is a transmembrane protein critical to cell adhesion (Reid and Hemperly 1990). Sher et al. indicated that CDH2 was the target gene of miR-218 for lung adenocarcinoma (Sher et al. 2014). The expression of vimentin, an intermediate filament protein, is associated with higher metastatic disease, and poor prognosis and survival of patients with multiple tumor types (Havel et al. 2015), including NSCLC patients (Soltermann et al. 2008; Dauphin et al. 2013). In addition, Havel et al. reported that vimentin affected lung cancer cell adhesion via a VAV2-Rac1 pathway to promote focal adhesion kinase stability (Havel et al. 2015). Our study demonstrated that miR-22-3p suppressed the EMT and invasion of NSCLC cells through the alteration of critical EMT markers, including E-cadherin, N-cadherin, and vimentin. These in vitro findings might explain why down-regulation of miR-22-3p was significantly correlated with NSCLC patients’ lymphatic metastasis status in clinic.

RAC1 is listed as a significant member of the Rho GTPases, which are involved in the tumor cell cycle, invasion, proliferation, apoptosis, angiogenesis, and migration, and are viewed as promising targets for preventing and treating cancers (Liang et al. 2021). RAC1 is a target of many miRNAs in various cancers, such as miR-194-5p in osteoclasts (Ni et al. 2021), miR‑142‑3p in colorectal cancer (Xie et al. 2021), and miR-509-3p in cervical cancer (Xu et al. 2021), miR-4715-5p in lung cancer (Yang et al. 2019). In lung cancer tissues, RAC1 expression was dramatically increased, and knockdown of RAC1 remarkably decreased cell migration, invasion, and proliferation. Akunuru et al. found that RAC1 knockdown with shRNA restrained the tumorigenic activities of human non-small cell lung adenocarcinoma (NSCLA) cells (Akunuru et al. 2011). Kaneto et al. reported that RAC1 inhibition could serve as a therapeutic target for gefitinib-resistant NSCLC (Kaneto et al. 2014). Tan et al. discovered RAC1 strengthened radioresistance by enhancing EMT through PAK1-LIMK1-Cofilins signaling in lung cancer, indicating that RAC1 may serve as a potential treatment target in radioresistant lung cancer cells (Tan et al. 2020). In this study, a new downstream target gene of miR-22-3p was discovered based on TargetScan, dual-luciferase reporter, RT-qPCR, and immunoblotting assays. The results showed that miR-22-3p directly interacted with the 3′-UTR of RAC1 mRNA, which resulted in reduced mRNA and protein expression of RAC1 in NSCLC cells. Importantly, inhibition of RAC1 contributing to the tumor-suppressive function of miR-22-3p because overexpression of RAC1 restored the migration and invasion ability of NSCLC cells inhibited by miR-22-3p mimics. Therefore, we demonstrated that miR-22-3p retarded the migration and invasion NSCLC cells through inhibition of RAC1’s expression via directly binding its 3′-UTR region.

There were several limitations in this study. (1) The clinical significance and biology function of miR-22-3p in small cell lung cancers (SCLCs) were not clarified. (2) Whether miR-22-3p/RAC1 axis suppressed the development of SCLC needed to be determined. In the future, we will perform experiments to illustrate the role and mechanisms of miR-22-3p, as well as other microRNAs in SCLC.

In conclusion, we provided the first evidence that RAC1, a well-known oncogene, acted as the direct downstream target of miR-22-3p in NSCLC. We demonstrated that miR-22-3p was down-regulated in NSCLC tissues and cells and suppressed cell migration and EMT via targeting RAC1. The miR-22-3p/RAC1 axis offers a novel therapeutic target for NSCLC.

## Data Availability

The datasets used and/or analyzed during the current study are available from the corresponding author on reasonable request.
